# The E3 Ubiquitin Ligase TRIM11 Facilitates Gastric Cancer Progression by Activating the Wnt/*β*-Catenin Pathway via Destabilizing Axin1 Protein

**DOI:** 10.1155/2022/8264059

**Published:** 2022-02-21

**Authors:** Ling Zhou, Heng Wang, Min Zhong, Zhi Fang, Yi Le, Fengting Nie, Juanjuan Zhou, Jianping Xiong, Xiaojun Xiang, Ziling Fang

**Affiliations:** ^1^Department of Oncology, The First Affiliated Hospital of Nanchang University, 1519 Dongyue Avenue, Nanchang, Jiangxi Province, China; ^2^Department of Orthopedics, The First Affiliated Hospital of Nanchang University, 1519 Dongyue Avenue, Nanchang, Jiangxi Province, China

## Abstract

**Background:**

Aberrant expression of tripartite motif 11 (TRIM11) and the Wnt/*β*-catenin pathway are essential for facilitating tumorigenesis and progression in multiple types of cancer.

**Aim:**

To investigate the molecular changes linking the dysregulation of TRIM11 and Wnt/*β*-catenin pathway activation in gastric cancer (GC) progression.

**Methods:**

The expression levels of TRIM11 were detected in GC tissues and cells by immunohistochemistry and western blotting. The role of TRIM11 in the growth, proliferation, and invasion of gastric cancer cells was observed by a series of cell functional experiments and further verified in vivo. Co-immunoprecipitation (Co-IP), immunofluorescence, cycloheximide, and western blotting assays and other experiments were conducted to explore the mechanisms of TRIM11 underlying the regulation of the Wnt/*β*-catenin pathway. For further verification, rescue experiments were performed by cotransfection of TRIM11 and Axin1 siRNA in GC cells.

**Results:**

Using Co-IP assays, we identified TRIM11 as a potent binding partner of Axin1 in GC cells. Elevated TRIM11 levels were significantly correlated with unfavorable clinical outcomes and poor survival in patients with GC. In addition, TRIM11 promoted the cell proliferation and invasion capacities of GC cells in vitro and tumor growth in vivo. Mechanistic investigations revealed that TRIM11 destabilized Axin1 protein by interacting with Axin1, thus inducing the activation of the Wnt/*β*-catenin pathway. Moreover, we found that the oncogenic effects of TRIM11 on GC cells were partly mediated by suppression of Axin1. Furthermore, the protein expression of TRIM11 and Axin1 was negatively correlated in GC tissues.

**Conclusion:**

Collectively, our findings not only establish a pivotal TRIM11-Axin1-*β*-catenin axis in driving GC progression but also indicate that TRIM11 serves as a valuable therapeutic target for the treatment of GC patients.

## 1. Introduction

Gastric cancer (GC) is one of the most prevalent malignancies worldwide, leading to a heavy burden on the society, especially in Asian countries [1]. Despite substantial progresses in surgical and comprehensive therapies, the survival and prognosis of advanced GC patients remain dismal [2, 3]. Therefore, it is imperative to explore the molecular mechanisms and signaling interactions that underlie GC tumorigenesis and progression.

A mounting number of studies have shown that activation of the Wnt/*β*-catenin signaling pathway is involved in multiple pathological processes, such as cell growth, cell cycle progression, invasion, and immune microenvironment [4–6]. When the canonical Wnt pathway is activated by the ligands Wnt1 and Wnt8, *β*-catenin accumulates in the cytoplasm and translocates to the nucleus, where it binds to TCF4/LEF, thereby inducing the transcription of downstream target genes, consequently leading to cell growth and metastasis [7]. In the absence of Wnt signaling, cytoplasmic *β*-catenin is phosphorylated by the destruction complex, which includes adenomatous polyposis coli (APC), Axin1/2, and glycogen synthase kinase-3*β* (GSK-3*β*). Then, phosphorylated *β*-catenin is recognized and degraded by *β*-transducin repeat-containing protein (*β*-TRCP) via the ubiquitin-proteasome system [8]. Recently, Axin1 has been identified as a critical negative regulator of the Wnt/*β*-catenin pathway in several tumors [9, 10]. However, the complex mechanisms underlying Axin1 dysregulation in GC remain largely elusive.

Tripartite motif 11 (TRIM11), a RING-type E3 ubiquitin-protein ligase, is reported to be involved in several human malignant processes, including exacerbated proliferation, metastasis, angiogenesis, and chemoresistance [11–13]. Previous studies have shown that TRIM11 is upregulated and associated with tumor growth and metastasis in glioma, lung cancer, hepatocellular cancer, and breast cancer [14–17]. Moreover, Tang et al. reported that the SOX13/TRIM11/YAP axis accelerates thyroid cancer cell proliferation, migration, and chemoresistance [18]. Signaling pathways underlying dysregulated TRIM11 function include the transduction nodes EGFR, Akt, and p53, which are highly related to oncogenesis [14, 19–21]. Recently, it has been demonstrated that TRIM11 is involved in the activation of the *β*-catenin signaling pathway via the EMT (epithelial-mesenchymal transition) process in GC [22]. Nevertheless, the exact correlations between TRIM11 and Wnt/*β*-catenin signaling in GC still need to be further elucidated.

In the present study, our data indicated that Axin1 functioned as an interacting protein of TRIM11. TRIM11 overexpression strengthened cell growth, migration, and invasion in GC cells, whereas TRIM11 depletion resulted in opposite effects. Mechanistically, TRIM11 shortened the half-life of Axin1 protein, thereby boosting the Wnt/*β*-catenin pathway. In summary, our findings provide new insights into the TRIM11-Axin1-*β*-catenin axis as an attractive therapy for GC tumorigenesis and progression.

## 2. Materials and Methods

### 2.1. Tissue Samples and Ethical Statement

A total of 150 paraffin-embedded GC tissues and paired adjacent noncancerous tissues with available clinical information were collected from the Department of Pathology at the First Affiliated Hospital of Nanchang University between 2014 and 2016. Similarly, eight pairs of fresh GC specimens and their corresponding adjacent tissues were obtained directly from the gastrointestinal operating room. All patients were treatment-naive before the surgery. The clinicopathological features were confirmed and summarized by two pathologists independently (Table 1 and Supplementary Table 1). This study was approved by the Ethics Committee of the First Affiliated Hospital of Nanchang University, and written informed consent was obtained from all patients.

### 2.2. Cell Lines and Cell Culture

The gastric cancer lines AGS, HGC-27, SGC-7901, BGC-823, MKN-45, MGC-803, and MKN-74 and the normal gastric epithelial cell line GES-1 were acquired from the Shanghai Institute of Cell Biology (Shanghai, China) or the American Type Culture Collection (ATCC). The GC cells were maintained in the RPMI-1640 medium or DMEM (both from Hyclone, Logan, USA) with 10%–15% fetal bovine serum (FBS, Biological Industries, BI, Israel) in a humidified chamber at 37°C in an atmosphere containing 5% CO_2_.

### 2.3. Western Blotting Analysis

Western blotting experiments were performed as previously reported [23]. Total protein extracted from cell or tissue lysates was treated with radioimmunoprecipitation assay lysis buffer containing a protease inhibitor cocktail (CoWin Biosciences, China). The primary antibodies used were as follows: TRIM11 (1 : 1000, Abcam, ab111694), Axin1 (1 : 500, CST, #2087), Axin2 (1 : 1000, Abcam, ab109307), *β*-catenin (1 : 1000, Proteintech, 51067-2-AP), cyclinD1 (1 : 1000, Abcam, ab239794), c-Myc (1 : 1000, Abcam, ab32072), and GAPDH (1 : 10000, Proteintech, 10494-1-AP). GAPDH was used as an internal control, and ImageJ software (https://imagej.net/ImageJ) was used to perform the densitometric analysis of the bands.

### 2.4. Immunohistochemistry (IHC)

The specimens were immunohistochemically stained using previously described methods [24]. Primary antibodies were diluted in the following proportions according to the manufacturer's instructions: TRIM11 (1 : 250; Abcam, ab111694) and Axin1 (1 : 50, CST, #2087). Immunohistochemical evaluation was performed by two independent pathologists using a microscope, and the score index (SI) values were obtained by multiplying the levels of staining (0, none; 1, weak; 2, moderate; 3, strong) and the positive range: 1 (0%–25%), 2 (26%–50%), 3 (51%–75%), and 4 (76%–100%). Clinical specimens with an SI ≥6 were defined as having high TRIM11 expression, while the rest of the samples were considered to have low TRIM11 expression.

### 2.5. RNA Isolation and qRT-PCR Assay

Total RNA from tissues and cells was isolated using TRIzol Reagent (Invitrogen; Thermo Fisher Scientific, Waltham, USA) and transcribed into cDNA using the PrimeScript kit (TransGen Biotech, Beijing, China) in accordance with the manufacturer's protocols as previously detailed [25]. Analysis of gene expression levels was performed using the SYBR^®^ Green kit (Bio-Rad, Hercules, USA) on a StepOnePlus system. The sequences of the primers used in the present study are included in Table 2. The relative expression of mRNA was calculated using the 2^−ΔΔCT^ method, and the expression levels of GAPDH were used as an internal control.

### 2.6. Vectors, Lentiviral Infection, and Transfection

Synthetic siRNAs targeting TRIM11 and Axin1 were purchased from GenePharma (Suzhou, China). The sequences of siRNAs were as follows: TRIM11 siRNA-1: 5′-CCAACCGCCCGCUUGCUAATT-3′, TRIM11 siRNA-2: 5′-GGAGAAGUCACUGGAGCAUTT-3′, and Axin1 siRNA: 5′-GGUAUGUGCAGGAGGUUAUTT-3′. The amplified human TRIM11, Axin1, and Axin2 sequences were subcloned into a pcDNA3.1 vector. The TRIM11 shRNA lentivirus plasmid (5-GGAGAAGTCACTGGAGCAT-3) was purchased from Jikai Gene Chemical Technology Company (Shanghai, China). Puromycin (final concentration: 2 *μ*g/mL) was used to select stable MKN-45 cells overexpressing TRIM11 for two weeks. All the transfection or infection experiments were performed using the transfection reagent TurboFect (Thermo Scientific, R0532, USA) as previously described [26, 27].

### 2.7. Cell Counting Kit-8 (CCK-8) and Colony Formation Assays

Cell viability was evaluated by CCK-8 and colony formation assays as previously described [27]. The GC cells were seeded in 96-well plates for the cell growth assay, with no less than three wells in each group. The cell growth rate was analyzed by measuring absorbance of each plate at 450 nm using a microplate reader for five consecutive days after seeding. For the colony formation assays, 1000 cells were seeded in six-well plates containing complete medium and grown for approximately 10–14 days. During this period, the medium was changed every 3-4 days. The colonies were then stained with 4% formalin and 1% crystal violet. ImageJ software was used to count the number of colonies. The cellular experiments were repeated in triplicate.

### 2.8. Transwell Assay

After trypsinizing the transfected cells, a serum-free cell suspension containing 3 × 10^3^ cells was prepared. Eight micrometer pore-size chambers (Corning Inc.) used in the transwell experiments were prepared with Matrigel gel (BD Biosciences) as previously described [28]. GC cells were inoculated into the upper chambers, and 500 *μ*L of the medium containing 15% FBS was added to the lower chamber as a chemical attractant. After 48–72 hours of incubation, the cells that had passed through the compartment membrane were fixed and stained for further analysis. Finally, the invading cells were observed and counted under a microscope in at least six randomly chosen fields. The experiments were performed using three biological replicates.

### 2.9. Wound Healing Assay

Scratch wound assays were used to test the migration capacity of GC cells after different treatments. GC cells were inoculated in a 6-well plate at a density of 4.5 × 10^5^ cells per well and then starved for 24 h until 90%–95% confluence was reached. Then, sterile 10 *μ*L pipette tips were used to scrape the bottom surface of the plate to form a wound vertically. Representative images were collected at 0 h and 48 h after the scratch, and the distance between the wound boundaries was measured to calculate the healing rate. The experiments were performed at least two times.

### 2.10. Cycloheximide (CHX) Chase Assay

The stability of Axin1 protein was examined using the CHX assay as previously described [26]. The cells were harvested and lysed at 0, 2, 4, 6, 8, and 10 h after the addition of CHX reagent (50 mg/mL). The lysates were subjected to western blotting analysis. The half-life decay curve of the protein was plotted using GraphPad Prism software.

### 2.11. Immunofluorescence Staining

Immunofluorescence experiments were performed according to the manufacturer's instructions. In brief, adherent cells were fixed in 4% paraformaldehyde at 4°C for 20 min and then washed thrice with PBS for 5 min. After washing, the cells were blocked with 5% skim milk in 0.1% Triton X-100 at room temperature for 1 h and then incubated with the primary antibodies at 4°C overnight. The next day, the cells were incubated with Alexa Fluor-labeled secondary antibodies for 1 h and washed three times with PBS buffer. Samples were incubated with 4′-6-diamidino-2-phenylindole (DAPI) for 30 min for nuclear staining. Finally, the cellular localization of TRIM11 and Axin1 was observed using a confocal microscope (Nikon, ECLIPSE Ti2), and representative images were captured and merged.

### 2.12. Co-Immunoprecipitation Assay

Co-IP assays were performed to investigate whether TRIM11 was a binding partner of Axin1/Axin2. In brief, as indicated in the figures, GC cells were cotransfected with plasmids encoding TRIM11 and Axin1 or Axin2 for 36 h and lysed to extract total protein. A total of 500–800 *μ*g of protein lysates was incubated with anti-FLAG beads (M8823, Sigma-Aldrich), anti-HA beads (B26201, Bimake), or anti-Myc (B26301, Bimake) at 4°C for 2–4 hours. The immunocomplexes were washed with lysis buffer at least three times and prepared for western blotting analysis using anti-FLAG (H7425, Sigma-Aldrich), anti-HA (H6908, Sigma-Aldrich), and anti-Myc (#06-340, Sigma-Aldrich) antibodies.

### 2.13. Tumor Xenograft Model In Vivo

BALB/C-nude female mice (5-6 weeks old) were obtained from Shanghai Laboratory Animal Company and randomly divided into two groups. A total of 1 × 10^6^ MKN-45 cells infected with TRIM11 shRNA or scramble shRNA were subcutaneously inoculated into the armpits. The tumor sizes of the mice were measured twice a week. The formula used for tumor size calculation was as follows: tumor volume (mm^3^) = 1/2 × (long diameter × short diameter^2^). Mice were sacrificed on day 28, and the xenograft tumors were excised, weighed, and photographed. The protein and RNA extracted from the tumor tissues were further analyzed by western blotting and qRT-PCR assays.

### 2.14. Statistical Analysis

Data are shown as the mean ± SEM. All biological experiments were conducted at least two times independently. The mean differences among groups were assessed using Student's two-tailed *t*-test or ANOVA. The survival rate was plotted and calculated using the Kaplan–Meier plotter using the log-rank test. The correlation of TRIM11 and Axin1 protein in GC tissues was evaluated using the Pearson correlation test. SPSS (version 26.0; IBM, USA) was used for statistical analysis. Statistical significance was set at *P* < 0.05.

## 3. Results

### 3.1. TRIM11 Interacts with Axin1

**Figure 1:**
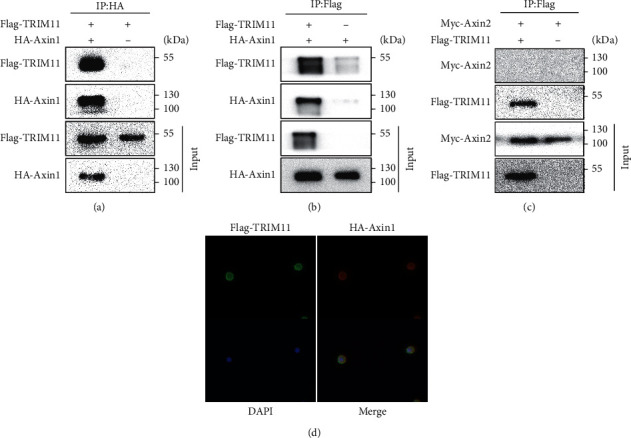
TRIM11 interacts with Axin1. (a, b) Co-immunoprecipitation assays were conducted to validate the exogenous binding of TRIM11 and Axin1 in GC cells. (c) Co-immunoprecipitation experiments were used to examine the interaction between TRIM11 and Axin2 in GC cells. (d) Immunofluorescence staining was used to observe the colocalization of TRIM11 and Axin1 in MKN-45 cells.

A recent study has shown that the ubiquitination of Axin1 by TRIM11 was involved in the carcinogenesis of lymphoma [29]. However, the detailed molecular mechanism underlying the involvement of TRIM11 and Wnt/*β*-catenin pathway in GC still needs to be further explored. Thus, we conducted co-immunoprecipitation and immunofluorescence assays to validate the interaction between TRIM11 and Axin1. Ectopic expression of FLAG-TRIM11 resulted in the complexes with HA-Axin1 (Figures 1(a) and 1(b)). However, we did not detect any interaction between TRIM11 and Axin2 (Figure 1(c)). Immunofluorescence analysis revealed that TRIM11 and Axin1 colocalized to the cytoplasm of GC cells (Figure 1(d)). These findings suggest that TRIM11 interacts with Axin1 in GC cells.

### 3.2. TRIM11 Is Highly Expressed and Predicts Disease Progression in GC

**Figure 2:**
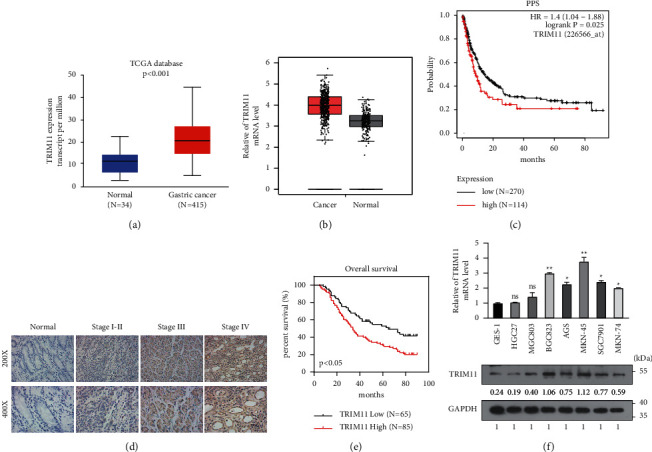
TRIM11 is highly expressed and predicts disease progression in gastric cancer (GC). (a, b) TRIM11 mRNA expression levels in GC tissues were investigated using UALCAN (https://ualcan.path.uab.edu/) (a) and GEPIA (https://gepia.cancer-pku.cn/) (b) websites. (c) The correlation between TRIM11 expression levels and clinical survival was analyzed using the Kaplan–Meier plotter online database (https://kmplot.com/analysis/). PPS: postprogression survival. (d) Immunohistochemical staining was performed to examine TRIM11 protein expression levels in GC tissues and adjacent nontumor gastric mucosa tissues. (e) Kaplan–Meier curve was plotted to explore the overall survival rate of enrolled GC patients using the log-rank test. (f) qRT-PCR and western blotting assays were performed to detect TRIM11 mRNA and protein levels in GC cell lines.

We further explored the expression patterns of TRIM11 in GC using the online tools UALCAN (https://ualcan.path.uab.edu/), GEPIA (https://gepia.cancer-pku.cn/), and Kaplan–Meier plotter (https://kmplot.com/analysis/). Online analysis revealed that TRIM11 expression was significantly elevated in GC tissues (Figures 2(a) and 2(b)) and that patients with lower expression levels of TRIM11 had better clinical outcomes than those with high TRIM11 expression levels (Figure 2(c)). To further verify the above results, we collected 150 paraffin-embedded specimens from GC patients undergoing surgical resection for immunohistochemical staining. As clearly shown in Figure 2(d), TRIM11 was overexpressed in cancerous tissues compared to the relevant normal tissues, and its expression tended to be higher as the TNM stage increased. Next, the follow-up data of these 150 patients were summarized to further assess the relationship between TRIM11 expression levels and clinicopathological features of gastric cancer patients. As illustrated in Table 1, the expression of TRIM11 was positively correlated with TNM stage (*P*=0.04), depth of invasion (*P*=0.014), and lymph node metastasis (*P*=0.015). Moreover, the Kaplan–Meier analysis indicated that the patients with high TRIM11 expression levels had worse overall survival than those with low TRIM11 expression levels (Figure 2(e)), indicating that TRIM11 may be a prognostic marker for GC patients. Consistently, the protein and mRNA expression levels of TRIM11 were increased in GC cell lines (Figure 2(f)). Collectively, our data indicate that TRIM11 is highly expressed and associated with disease progression in GC.

### 3.3. TRIM11 Strengthens the Proliferation and Invasion Capacities of GC Cells

**Figure 3:**
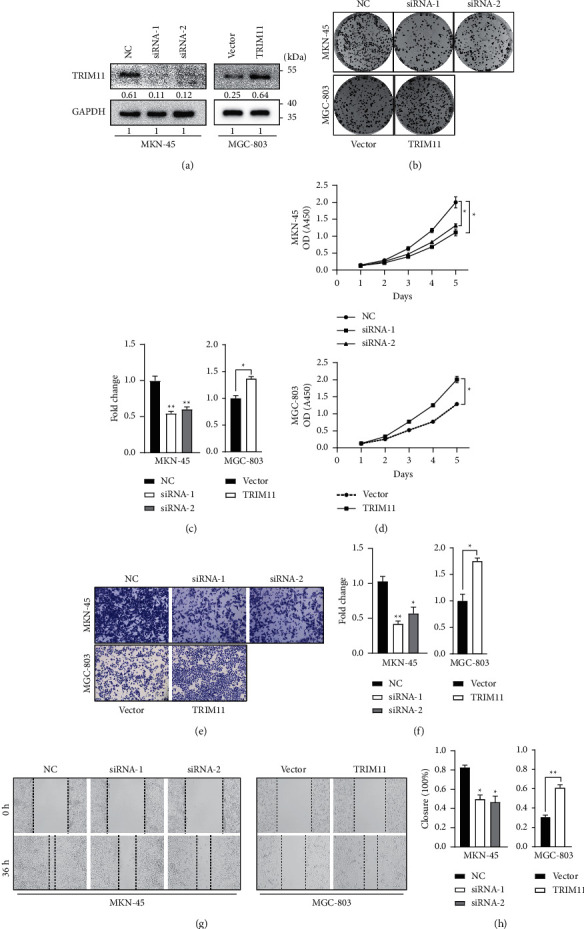
TRIM11 strengthens the proliferation and invasion capacities of gastric cancer (GC) cells. (a) Transfection efficiency of TRIM11 siRNAs and overexpression plasmids was confirmed using western blotting in GC cells. (b, c) Representative photograph of colony-forming assays for GC cells; the bar graph shows the quantification of fold changes. (d) CCK-8 assays were used to detect cell viability upon TRIM11 overexpression or knockdown. (e, f) Representative images and quantification of the cells invaded into the lower chamber upon indicated transfection compared with the negative control group. (g, h) Wound healing assays were utilized to assess the migratory capacities of GC cells upon TRIM11 regulation (^*∗*^*P* < 0.05 and ^*∗∗*^*P* < 0.01 vs. the corresponding control groups).

We also investigated the biological functions of TRIM11 in vitro using overexpression and knockdown assays. Transfection efficiency was validated by western blotting analysis (Figure 3(a) and Supplementary Figures 1(a) and 1(i)). In terms of the results of colony formation and CCK-8 assays, the cell growth of MKN-45 cells was suppressed upon TRIM11 depletion (Figures 3(b)–3(d) and Supplementary Figures 1(b)–1(d), *P* < 0.05). In contrast, upregulation of TRIM11 substantially promoted cell proliferation abilities in GC cells (Figures 3(b)–3(d) and Supplementary Figures 1(j)–1(l), *P* < 0.05). Additionally, the wound healing and transwell invasion assays revealed that TRIM11 knockdown resulted in reduced migratory and invasive abilities (Figures 3(e)–3(h) and Supplementary Figures 1(e)–1(h), *P* < 0.05), while TRIM11 overexpression showed the opposite effects (Figures 3(e)–3(h) and Supplementary Figures 1(m)–1(p), *P* < 0.05). Overall, these results indicate that TRIM11 plays an essential role in accelerating the proliferation and metastasis of GC cells.

### 3.4. TRIM11 Activates the Wnt/*β*-Catenin Signaling Pathway by Destabilizing Axin1 Protein

**Figure 4:**
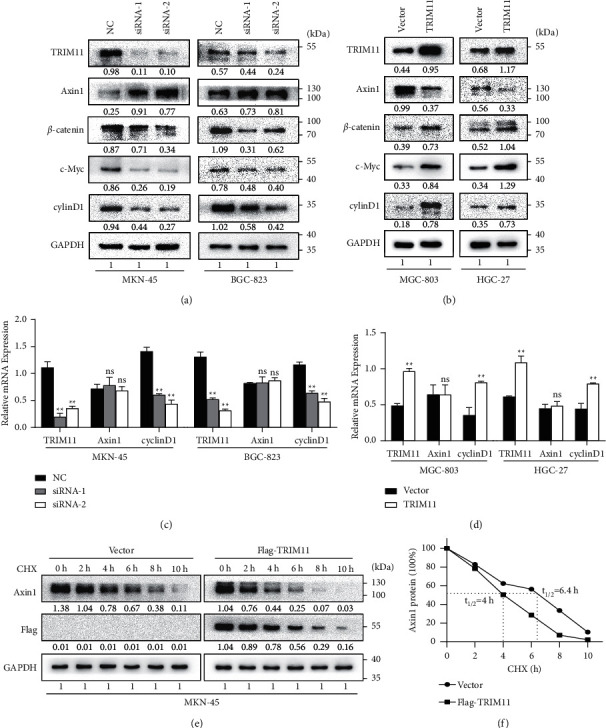
TRIM11 activates the Wnt/*β*-catenin signaling pathway by destabilizing Axin1 protein. (a, b) Protein expression of TRIM11 and Wnt/*β*-catenin cascade components was determined using western blotting assays in GC cells upon indicated transfection. (c, d) The mRNA levels of Wnt/*β*-catenin pathway components were assessed using qRT-PCR assays upon TRIM11 knockdown or overexpression in GC cells. (e) Representative western blotting images of Axin1 protein in MKN-45 cells treated with 50 mg/ml cycloheximide (CHX) for 0, 2, 4, 6, 8, and 10 h. (f) Line graph displays Axin1 protein expression over time normalized to GAPDH controls. ns: no significance (^*∗*^*P* < 0.05 and ^*∗∗*^*P* < 0.01 vs. the corresponding control groups).

Previous studies have demonstrated that Axin1/2 interacts with GSK-3*β* and APC, forming the destruction complex that inactivates the *β*-catenin signaling pathway [30–32]. Therefore, we further intended to clarify how the TRIM11-Axin1 interaction affects the Wnt/*β*-catenin pathway. As shown in Figures 4(a) and 4(b), the core components of the Wnt pathway, *β*-catenin, c-Myc, and cyclinD1 were significantly decreased upon TRIM11 knockdown, whereas overexpression of TRIM11 had the opposite effects. However, the qRT-PCR assays indicated that Axin1 mRNA was not significantly changed upon TRIM11 knockdown or upregulation (Figures 4(c) and 4(d)), indicating that the regulation of Axin1 by TRIM11 occurred at the posttranscriptional level. Therefore, we visualized the degradation kinetics of Axin1 protein using the cycloheximide (CHX) chase assay and found that the half-life of Axin1 protein was significantly shortened upon TRIM11 overexpression (6.4 h vs. 4.0 h, *P* < 0.05, Figures 4(e) and 4(f)). Altogether, these data indicate that TRIM11 orchestrates the Wnt/*β*-catenin signaling pathway by destabilizing the Axin1 protein.

### 3.5. Axin1 Silencing Rescues the Cancer-Suppressing Roles Mediated by TRIM11 Depletion in GC Cells

**Figure 5:**
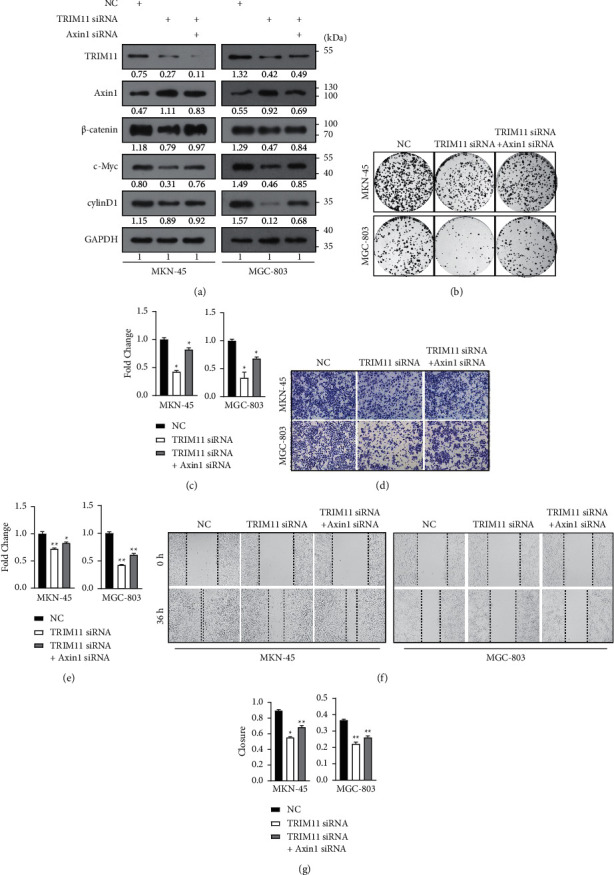
Axin1 silencing rescues the cancer-suppressing roles of TRIM11 depletion in gastric cancer (GC) cells. (a) Western blotting analysis was performed to detect the protein expression levels of TRIM11, Axin1, and Wnt/*β*-catenin core components upon Axin1 silencing. (b–g) The proliferation, invasive, and migration abilities of MKN-45 and MGC-803 cells were determined by colony formation (b, c), transwell invasion (d, e), and wound healing assays (f, g) (^*∗*^*P* < 0.05 and ^*∗∗*^*P* < 0.01 vs. the corresponding control groups).

The gain-of-function assays were used to ascertain whether Axin1 silencing could abolish the effects of TRIM11 depletion in GC cells. As expected, Axin1 depletion partially abolished the downregulation of *β*-catenin, c-Myc, and cyclinD1 induced by TRIM11 knockdown (Figure 5(a)). In addition, knockdown of Axin1 largely abrogated the suppression of the colony formation ability observed in GC cells when TRIM11 was silenced (Figures 5(b) and 5(c), *P* < 0.05). Similar phenotypes were also detected using the wound healing and transwell assays (Figures 5(d)–5(g), *P* < 0.05). Collectively, these data support that Axin1 is an essential contributor to TRIM11-mediated GC cell proliferation and metastasis.

### 3.6. TRIM11 Depletion Inhibits Tumor Growth via Regulating the *β*-Catenin Pathway In Vivo

**Figure 6:**
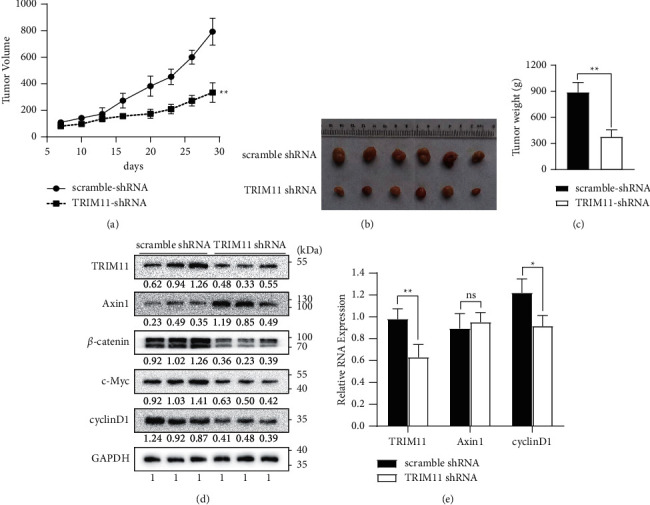
TRIM11 depletion inhibits tumor growth via regulating the *β*-catenin pathway in vivo. (a) Tumor volume growth curve of the negative control mice and TRIM11-knockdown mice. (b, c) Xenograft tumors were harvested on the 28th day after injection. The weights of the tumors obtained from the two groups are shown in the bar graph. (d, e) Protein expression levels of TRIM11, Axin1, and Wnt pathway core components were analyzed in xenograft tumors. (f) The mRNA expression levels of TRIM11, Axin1, and cyclinD1 in six harvested tumors were detected by qRT-PCR assays. ns: no significance (^*∗*^*P* < 0.05 and ^*∗∗*^*P* < 0.01 vs. the corresponding control groups).

To elucidate the impact of TRIM11 on GC tumorigenesis more credibly, we constructed a xenograft model by injecting TRIM11-knockdown MKN-45 cells in the armpit of BALB/C-nude mice and measured the tumor size for 28 days. The injection of TRIM11-knockdown MKN-45 cells leads to a reduced tumor growth and tumor weight in comparison with the injection of control MKN-45 cells (Figures 6(a)–6(c), *P* < 0.05). In line with previous cellular experiments, western blotting analysis of the xenograft tissues showed that TRIM11 knockdown markedly increased Axin1 protein expression levels, thus causing inactivation of the Wnt/*β*-catenin pathway (Figure 6(d)). Consistently, the mRNA levels of TRIM11 and cyclinD1 were greatly decreased. However, no significant changes in Axin1 mRNA expression were observed upon TRIM11 depletion (Figure 6(e)). Collectively, these results indicate that TRIM11 depletion inhibits tumor growth by regulating the *β*-catenin pathway in vivo.

### 3.7. TRIM11 Expression Is Inversely Correlated with Axin1 Expression in GC Tissues

**Figure 7:**
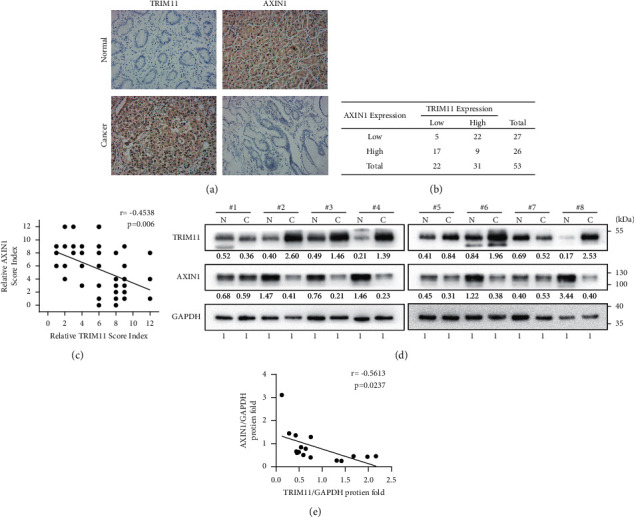
TRIM11 expression is inversely correlated with Axin1 expression in GC tissues. (a) Representative immunohistochemistry staining of TRIM11 and Axin1 protein expression in GC tissues. Summary of immunohistochemical scores (b) and correlation analysis of TRIM11 and Axin1 expression (c) in 53 GC patients. (d) TRIM11 and Axin1 protein expression levels in eight paired GC tissues and adjacent normal tissues were analyzed by western blotting. (e) Correlation analysis of TRIM11 and Axin1 expression levels in eight GC patients using ImageJ software. Pearson's correlation coefficient was analyzed using the chi-square test.

Immunohistochemical staining was performed to illustrate the relationship between TRIM11 and Axin1 expression in clinical GC specimens. Consistent with the aforementioned cellular experiments, TRIM11 protein levels were frequently elevated, while Axin1 was downregulated in cancerous tissues when compared to adjacent normal tissues (Figure 7(a)). Statistical analysis showed an inverse relationship between TRIM11 and Axin1 protein expression levels (Figures 7(b) and 7(c), *P*=0.006, *r* = −0.4538). Moreover, TRIM11 was markedly upregulated in most fresh GC tissues (6/8, 75%; Figure 7(d)) and was negatively correlated with Axin1 protein expression levels (Figure 7(e), *P*=0.0237, *r* = −0.5613). These results suggest that TRIM11 protein expression is inversely correlated with Axin1 expression in GC tissues.

## 4. Discussion

Our study showed that TRIM11 was drastically overexpressed in GC and that its upregulation was associated with poor outcomes and shorter survival time in GC patients. Additionally, TRIM11 functioned as an oncogene in GC cells, and its cancer-promoting effects were mainly mediated by the Axin1-*β*-catenin axis. To the best of our knowledge, we demonstrated for the first time that TRIM11 could bind to Axin1 and decrease Axin1 protein stability, thereby resulting in the activation of the Wnt/*β*-catenin pathway in GC cells. Therefore, our study not only provides new insights into the intricate regulatory network of the Wnt/*β*-catenin pathway but also demonstrates that the TRIM11-Axin1-*β*-catenin axis favors GC carcinogenesis and development.

There are compelling literature studies reporting that the Wnt/*β*-catenin signaling pathway is one of the most predominant pathways involved in gastric oncogenesis [33]. Axin1, which functions as a concentration-limiting component of the *β*-catenin destruction complex, has been shown to negatively regulate the Wnt/*β*-catenin pathway in several cancers [34]. In this study, we first identified that TRIM11 served as a novel Axin1-binding protein and expanded the role of TRIM11 in GC. Previously, TRIM11 upregulation had been detected in a series of human cancers, such as pancreatic ductal adenocarcinoma, renal clear cell carcinoma, ovarian cancer, and breast cancer [11, 20, 35]. Luo and Wang showed that TRIM11 upregulation was positively correlated with advanced pathological stage, large tumor size, and poor prognosis in GC [19]. In line with these results, our results showed that TRIM11 was elevated in most of the GC cases (56.67%) and associated with poor clinical outcomes. In contrast to their report [22], our study revealed that TRIM11 protein expression was an indicator of lymph node metastasis in GC patients. Moreover, we demonstrated that TRIM11 depletion significantly inhibited cell growth and invasion, whereas these were enhanced upon TRIM11 overexpression. These findings further indicate that TRIM11 is a potential oncogene, suggesting that it may be a therapeutic target for GC treatment in the future. However, the molecular mechanisms by which TRIM11 exerts oncogenic effects require further investigation.

Several studies have shown that Axin1 protein expression is frequently regulated by proteasomal degradation and that inhibition of Axin1 results in the activation of the Wnt/*β*-catenin pathway [36, 37]. Moreover, it has been reported that TRIM11 is involved in the activation of the *β*-catenin pathway via the ubiquitination and degradation of Axin1 in lymphomas [29] and that it promotes cell growth and epithelial-mesenchymal transition in gastric cancer by activating *β*-catenin signaling [22]. Here, we demonstrated for the first time that TRIM11 binds to Axin1, thus shortening the half-life of Axin1 and leading to activation of the Wnt/*β*-catenin pathway. TRIM11 overexpression increased the expression of Wnt pathway target genes, including c-Myc and cyclinD1. More intriguingly, the regulatory role of TRIM11 in biological functions could be partially rescued by Axin1 overexpression, further clarifying that Axin1 acts as a predominant mediator in the TRIM11-mediated regulation of the Wnt/*β*-catenin pathway in GC cells. However, it should be noted that TRIM11-induced effects could not be completely abolished by Axin1 upregulation, indicating that Axin1 is not the unique mediator between TRIM11 and the Wnt/*β*-catenin pathway. As TRIM11 has been reported to also regulate Axin2, USP14, PHLPP1/Akt, and EGFR [15, 38–40], we hypothesize that TRIM11 may also modulate these oncogenes and pathways to stimulate the Wnt pathway in GC cells. More interestingly, a significant negative correlation was found between the TRIM11 and Axin1 protein expression levels in GC tissues, further supporting that TRIM11 upregulation might contribute to the activation of the Wnt/*β*-catenin pathway through the suppression of Axin1 expression. Besides, some GC patients presented low TRIM11 and Axin1 expression levels simultaneously, suggesting that Axin1 could also be downregulated by other mechanisms, such as by the protein YTHDF2 or by miRNAs at the transcriptional or posttranscriptional level [41, 42]. Altogether, our findings not only provide new insights into the regulation of Axin1 expression in GC but also present novel strategies for therapeutics targeting the Wnt/*β*-catenin pathway.

## 5. Conclusions

In summary, our study defines TRIM11 as a bona fide activator of the Wnt/*β*-catenin pathway to facilitate GC tumorigenesis and progression by destabilizing Axin1 protein. Induction of TRIM11 is a novel therapeutic choice to suppress Axin1 expression and orchestrate *β*-catenin activation, thus inhibiting the Wnt pathway-driven GC tumorigenesis and progression.

## Figures and Tables

**Table 1:** The relationship between TRIM11 and clinical factors of gastric cancer patients.

Clinicopathological parameters	*N*	TRIM11 expression (%)		*P* value
		Negative	Positive	
	150	65 (43.3)	85 (56.7)	
Age (years)				
<65	93	40 (43.0)	53 (57.0)	0.919
≥65	57	25 (43.9)	32 (56.1)	
Sex				
Male	87	39 (44.8)	48 (55.2)	0.664
Female	63	26 (41.3)	37 (58.7)	
Tumor size (cm)				
<5	78	39 (50.0)	39 (50.0)	0.086
≥5	72	26 (36.1)	46 (63.9)	
Differentiation				
Moderately or well	71	35 (49.3)	36 (50.7)	0.162
Poorly	79	30 (38.0)	49 (62.0)	
Depth of invasion				
T1-T2	66	36 (54.5)	30 (45.5)	0.014
T3-T4	84	29 (34.5)	55 (65.5)	
Lymph node metastasis				
With	64	35 (54.7)	29 (45.3)	0.015
Without	86	30 (34.9)	56 (65.1)	
TNM stage				
I-II	62	33 (53.2)	29 (46.8)	0.040
III-IV	88	32 (36.4)	56 (63.6)	
Tumor location				
Proximal	65	30 (46.2)	35 (53.8)	0.542
Distal	85	35 (41.2)	50 (58.8)	
Lauren classification				
Intestinal type	91	43 (47.3)	48 (52.7)	0.229
Diffuse type	59	22 (37.3)	37 (62.7)	

All the factors were analyzed by the chi-square test.

**Table 2:** The sequences of primers utilized in this study for quantitative real-time PCR analysis.

Target gene	Primers
TRIM11	Forward: 5′-CGAAGACCTCAAGGCGAAGC-3′
	Reverse: 5′-CAGCAAACGGCGAAGACG-3′

Axin1	Forward: 5′-ACAGCATCGTTGTGGCGTAC-3′
	Reverse: 5′-CAGTCAAACTCGTCGCTCAC-3′

CyclinD1	Forward: 5′- CCCTCGGTGTCCTACTTCAA-3′
	Reverse: 5′- GGGGATGGTCTCCTTCATCT-3′

GAPDH	Forward: 5′-AATCCCATCACCATCTTC-3′
	Reverse: 5′-AGGCTGTTGTCATACTTC-3′

## Data Availability

The datasets performed or analyzed in this work are available from the corresponding authors Ziling Fang and Xiaojun Xiang upon reasonable request.

## Abbreviations

GC: Gastric cancer
TRIM11: Tripartite motif 11
qRT-PCR: Quantitative reverse transcription PCR
CCK-8 assay: Cell Counting Kit-8 assay
CHX: Cycloheximide.

## Supplementary Materials

Supplementary Materials Supplementary Figure 1: TRIM11 regulates cell proliferation, migration, and invasion abilities of GC cells. Supplementary Table 1: the clinicopathological features of 8 GC patients.
