# Supplementary material for: The E3 Ubiquitin Ligase TRIM11 Facilitates Gastric Cancer Progression by Activating the Wnt/β-Catenin Pathway via Destabilizing Axin1 Protein
Source: J Oncol. 2022 Feb 21;2022:8264059. doi: 10.1155/2022/8264059 (PMC8885197; doi:10.1155/2022/8264059)
Supplement: Supplementary Materials — Supplementary Figure 1: TRIM11 regulates cell proliferation, migration, and invasion abilities of GC cells. Supplementary Table 1: the clinicopathological features of 8 GC patients. [file 8264059.f1.zip › 8264059.f1/Supplementary Figure 1.docx]

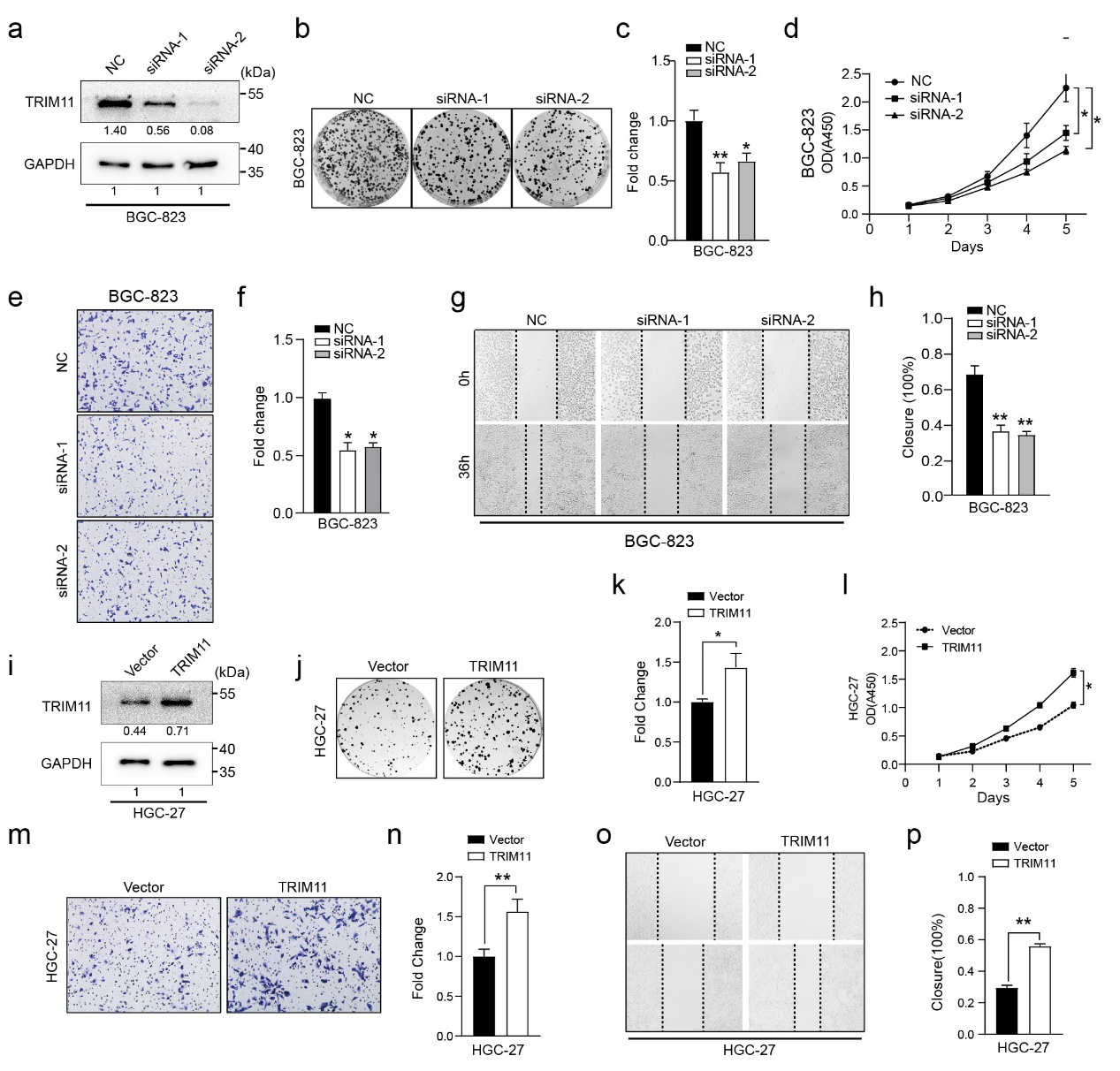


S_upplementary Figure_ 1: TRIM11 regulates cell proliferation, migration and invasion abilities of GC cells.

(a) Transfection efficiency of TRIM11 siRNAs was validated using western blotting in BGC-823 cells. (b, c) The clonogenic ability of GC cells was determined using the colony formation assays. (d) CCK-8 assays were conducted to detect the cell growth rate. (e, f) Representative images of the invaded cells were shown. (g, h) Scratch wound assays were used to examine the migrative capacities of BGC-823 cells upon TRIM11 knockdown. (i) The transfection efficiency of the TRIM11 overexpression plasmid was assessed using western blotting experiments in HGC-27 cells. (j-p) colony formation (j, k), CCK-8 (l), transwell invasion (m, n), and wound healing assays (o, p) were used to evaluate the proliferation, migration and invasion capacities of HGC-27 cells upon TRIM11 overexpression. (*P < 0.05; **P < 0.01 vs. the corresponding control groups).
