# Supplementary material for: The E3 Ubiquitin Ligase TRIM11 Facilitates Gastric Cancer Progression by Activating the Wnt/β-Catenin Pathway via Destabilizing Axin1 Protein
Source: J Oncol. 2022 Feb 21;2022:8264059. doi: 10.1155/2022/8264059 (PMC8885197; doi:10.1155/2022/8264059)
Supplement: Supplementary Materials — Supplementary Figure 1: TRIM11 regulates cell proliferation, migration, and invasion abilities of GC cells. Supplementary Table 1: the clinicopathological features of 8 GC patients. [file 8264059.f1.zip › 8264059.f1/Supplementary Table 1.docx]

**Supplementary table 1: The clinicopathological features of 8 GC patients.**

| **Patients** | **Age (year)** | **gender** | **Tumor size (cm)** | **Differentiation (well, moderate and poorly)** | **Depth of invasion** | **TNM stage** | **Lymph node metastasis**  **(N_0_ or N_X_)** |
| --- | --- | --- | --- | --- | --- | --- | --- |
| #1 | 67 | Male | 6*3 | Moderate | T2 | IIB | N3 |
| #2 | 55 | Male | 3.2*2.7*1.5 | Moderate | T4 | IIIC | N3 |
| #3 | 62 | Male | 6*4*1.5 | Moderate | T4 | IIIC | N3 |
| #4 | 64 | Male | 2.8*2.2*0.8 | poorly | T4 | IIIA | N1 |
| #5 | 86 | Male | 6*4.5*1.8 | Moderate | T4 | IIIC | N3 |
| #6 | 56 | Male | 3*3*1 | poorly | T4 | IIIC | N3 |
| #7 | 55 | Male | 2.5*2*0.3 | Moderate | T2 | IIA | N1 |
| #8 | 92 | Male | 3.5*4*2.5 | Moderate | T4 | IIIB | N2 |

The TNM stage of the patients was classified in terms of the AJCC staging system (8^th^ edition, 2016) for GC.
